# Analytical and Numerical Treatments of Conservative Diffusions and the Burgers Equation

**DOI:** 10.3390/e20070492

**Published:** 2018-06-25

**Authors:** Dimiter Prodanov

**Affiliations:** Department of Environment, Health and Safety, Imec, 3001 Leuven, Belgium; dimiter.prodanov@imec.be

**Keywords:** stochastic differential equations, Monte Carlo simulations, Burgers equation, Langevin equation, fractional velocity, 02.30.Jr, 02.30.Uu, 02.50.Ga, 02.70.Uu, 60J65, 76R50, 65R20

## Abstract

The present work is concerned with the study of a generalized Langevin equation and its link to the physical theories of statistical mechanics and scale relativity. It is demonstrated that the form of the coefficients of the Langevin equation depends critically on the assumption of continuity of the reconstructed trajectory. This in turn demands for the fluctuations of the diffusion term to be discontinuous in time. This paper further investigates the connection between the scale-relativistic and stochastic mechanics approaches, respectively, with the study of the Burgers equation, which in this case appears as a stochastic geodesic equation for the drift. By further demanding time reversibility of the drift, the Langevin equation can also describe equivalent quantum-mechanical systems in a path-wise manner. The resulting statistical description obeys the Fokker–Planck equation of the probability density of the differential system, which can be readily estimated from simulations of the random paths. Based on the Fokker–Planck formalism, a new derivation of the transient probability densities is presented. Finally, stochastic simulations are compared to the theoretical results.

## 1. Introduction

The Langevin equation was introduced in order to describe the motion of a test particle subjected to a fluctuating force and a viscous drag [1]. Its formulation was later generalized to encompass also other types of systems. The Langevin equation is also fundamental for the stochastic interpretation of Quantum Mechanics (QM) [2] and it also appears, in the form of a geodesic equation, in the scale relativity theory (SR) developed by Nottale [3]. The equation represents a substantial theoretical innovation because it was in fact the first stochastic differential equation. The formal theory of stochastic differential equations was developed much later by the works of Itô and Stratonovich (see, for example, [4] for introduction).

In contrast to the picture of diffusion as an uncorrelated random walk, the theory of dynamical systems makes it possible to treat diffusion as a deterministic dynamical process. There the Langevin dynamics can be also driven by chaotic but deterministic processes [5,6,7]. Emergence of diffusive behavior and Markovian evolution was also addressed by Gillespie [8]. The recent study of Tyran-Kaminska demonstrates that simple diffusion processes can emerge as weak limits of piecewise continuous processes constructed within a totally deterministic framework [7]. This is a finding which lends credence to the widely used techniques of Monte Carlo simulations using pseudo-random number generators.

A different way of looking at the Langevin equation is to specify a *fractal* driving process instead of the stochastic Wiener process. Examples can be given by the studies of deterministic diffusion, where generalized Takagi functions appear [9,10]. Using this approach, both fractal and linear behaviours of the diffusion coefficients can be demonstrated. Together, the studies mentioned so far demonstrate a fundamental interplay between emergent stochasticity, chaotic dynamics and fractality, which governs transport phenomena.

The term *generalized Langevin equation* is typically used in the physics literature to describe the system’s memory effects conveyed by non-Markovian color noises [11]. The present paper will generalize the Langevin equation in a different way. The Markovian character of the driving signal will be preserved, but the signal will be assumed to have some properties, leading to fractal behaviour—notably a suitably dense set of points where its Hölder exponent is fractional. Furthermore, the linearity restriction of drift term will be relaxed and instead the drift will be assumed to be a smooth function of position and time.

Interpretations of quantum mechanics are drawing a reemerging attention in the light of the centennial anniversary of David Bohm. Part of the present work was presented as a poster at the Emergent Quantum Mechanics 2017 conference in London. Results of the present work have been derived using the machinery of stochastic mechanics. On the other hand, the paper does not make strong foundational claims; instead, it is concerned with some questions about the mathematical foundations of the scale relativity theory, its link to stochastic mechanics and the theory of the Burgers equation. To the author’s knowledge, such a link to the Burgers equation was not recognized before.

The Burgers equation was initially formulated by Bateman while modeling the weakly viscous liquid motion [12]. It can be derived from the full Navier–Stokes equations under some simplifying assumptions. It was later studied extensively by Burgers as a cartoon model of turbulence [13]. Presently, the number of applications of the Burgers equation is very diverse. It has been used to model physical systems, such as surface perturbations, acoustic waves, electromagnetic waves, density waves, or traffic (see, for example, [14]). The stochastic representation of the Burgers equation can be traced back to the seminal works of Busnello et al. [15,16]. Later, Constantin and Iyer derived a probabilistic representation of the deterministic three-dimensional Navier–Stokes equations [17,18]. The result presented here complements the findings of these authors as incompressibility, and hence the harmonicity of the drift, in the Burgers equation is not required.

The paper starts by briefly presenting stochastic mechanics and scale relativity. Section 2 demonstrates a general result about stochastic representations of Hölder-continuous signals leading to the Langevin equation. Section 3 introduces Nelson’s characterization of a stochastic process. Section 4 introduces the complex representation of the drift in stochastic mechanics and scale relativity. Section 5 establishes the connection with the Burgers equation. Based on the Fokker–Planck formalism, a new derivation of the transient probability densities is presented. Section 6 discusses the Burgers equation as a geodesic-type of equation. The Cole–Hopf transformations are discussed as solution techniques for the Burgers equation in Section 7. Moreover, it is demonstrated how complex Cole–Hopf transformations map the complex Burgers equation, derived in a variational setting, to the free Schroedinger equation. Finally, in Section 8, numerical simulations are compared with the theoretical results.

### 1.1. Stochastic Mechanics (SM)

In the 1930s, certain similarities between the equations of classical statistical mechanics and the Schrödinger equation were discovered. These findings led to the stochastic interpretation of quantum mechanics. In stochastic mechanics, quantum phenomena are described in terms of diffusions instead of wave functions. The main equation of motion is in fact the Langevin equation. The formal equations of stochastic mechanics were formulated at first by Fényes [19] and Weizel [20] and later taken up by Nelson [2]. Following this interpretation, the trajectories of the configuration, described by a Markov stochastic process, are regarded as physically real. Nelson’s original formulation employed a stochastic version of the Newton’s law and time reversibility of the process. Interestingly, the form of the stochastic acceleration had to be postulated.

A Lagrangian formulation of stochastic mechanics was achieved by Pavon in complex form [21]. However, the given presentation is far from intuitive. In his treatment, the stochastic Lagrangian is the classical Lagrangian evaluated on a complex-valued velocity field in place of the real-valued classical velocity, while the dynamics is given by a complex-valued stochastic differential equation, similar to the treatment of Nottale. The Lagrangian problem was formulated as a constrained optimization problem, where the dynamics acted as the constraint.

### 1.2. Scale Relativity Theory (SR)

The scale relativity theory extends the principle of relativity also to resolution scales [3,22,23]. The main tenet of the theory is that there is no preferred scale of description of the physical reality. Therefore, a physical phenomenon must be described simultaneously at all admissible scales. While this is consistent with calculus for differentiable signals, the situation changes if non-differentiable models, such as Brownian motion or Mandelbrot’s multiplicative cascades [24], are addressed. For these cases, the scale of observation (or resolution) is present irreducibly in the local description of a phenomenon. This led Nottale to postulate the fractality of the underlying mathematical variety (i.e., a pseudo-manifold) describing the observables. It should be noted that, in Nottale’s approach, only finite differences are admissible. The scale relativistic approach results in corrections of Hamiltonian mechanics that arise due to the non-differentiability of trajectories, which are treated as virtual paths. Nottale introduces a complex operator that he calls the scale derivative, which acts as a pseudo-derivative (see Section 4 for details).Using this tool, Nottale gives an informal derivation of the Schrödinger equation from the classical Newtonian equation of dynamics, via a quantization procedure that follows from an extension of Einstein’s relativity principle called the scale relativity principle.

## 2. Stochastic Representation of Trajectories

If one considers the Brownian particle as a subsystem and the surrounding particles as an infinite dimensional thermal reservoir, the Langevin equation precisely models the situation where the subsystem suitably interacts with the thermal reservoir. The type of the effective random force can be identified with a Wiener process, which has continuous but non-differentiable paths almost everywhere. Mathematical descriptions of strongly nonlinear phenomena necessitate the relaxation of the global assumption of differentiability. In contrast, classical physics assumes global smoothness of the signals and continuity of their first two derivatives. Therefore, non-smooth phenomena, such as fractals slip through its conceptual net. This argument can be further elaborated as follows. Consider the measurement of a trajectory in time x(t). Non-differentiability can occur in three scenarios:

1. divergence of the velocity, that is divergence of the difference quotient,
2. oscillatory singularity or
3. difference between forward and backward velocities.

While for scenarios (1) and (2) the velocities (i.e., derivatives) can not be defined mathematically, scenario (3) requires dropping only the assumption of continuity of the resulting velocity. That is, $x_{+}^{\prime }\left(t\right)\neq x_{-}^{\prime }\left(t\right)$ at the point of non-differentiability *t*. A simple example of such behavior is the signal x(t)=|t| around the origin t=0. While scenario (2) is excluded by the scale relativity theory, scenario (1) leads to scale dependence of the difference quotient. Examples of fractal functions, such as the mathematical Brownian motion paths, are typically of divergent length. This at best can be viewed as a mathematical idealization since in this case the work for moving a particle along its trajectory must be infinite. On the other hand, non-differentiability does not need to occur “everywhere” (i.e., with full Lebesgue measure) on a trajectory. In this case, the trajectory can be almost everywhere differentiable except on a certain dense set of points. Examples of these are the singular functions, such as the Salem-de Rham’s functions [25] or the well known Cantor’s function. Singular functions have finite lengths, therefore the exerted displacement work is also finite. This makes them promising candidates for conceptualization of non-smooth phenomena in physics.

The relationship between Nelson’s and Nottale’s approaches can be established in a formal way. For clarity of the argument, we focus on the one-dimensional case. First, let’s establish the concept of stochastic embedding of a signal. In the following, we assume that the deterministic signal (i.e., trajectory) will be represented by an **equivalence class of stochastic paths** having the same expectation as the given deterministic signal. Mathematical notation and preliminaries for the subsequent treatment are presented in Appendix A. A possibly non-differentiable continuous trajectory is represented by a continuous Markov stochastic process evaluated in the virtual space of paths as follows:

**Definition** **1** (Markov Stochastic Embedding)**.** *Consider a bounded deterministic signal x(t) on the compact interval $T\subseteq R^{\;}$ representing time. Define the stochastic embedding $S_{\rho }$ in the probability space (T⊗Ω,F,ρ), where ρ is the probability density, as the isomorphism*

$$\begin{array}{cc} S_{\rho }: & \;T⊗R^{\;}\mapsto \left(T⊗\Omega ,F,\rho \right),\\ S_{\rho }: & \;(t,x(t))\mapsto X(t,\omega ),\;X\in T⊗\Omega , \end{array}$$

*under the constraint*

$$E^{\omega }\;X\left(t,\omega \right)=x\left(t\right),$$

*where the random variables sampled at different times t are independent and identically distributed (i. i. d.) and F is a σ-algebra.*

Note: the ω-index will be skipped from the notation wherever convenient for clarity. In addition, $X_{t}$ and X(t) will be used interchangeably. Deterministic signals are denoted by the lower case, while the stochastic by upper case letters.

The above definition implicitly assumes that $X_{t}\in L^{1}\left(T⊗\Omega ,F,\rho \right)$ and $E^{\omega }\;X\left(t,\omega \right)<\infty$.

The name of the embedding is justified by the following Lemma:

**Lemma** **1.** The stochastic process under the above definition has the Markov property.

**Proof.**  By construction for fixed t,δ∈F

$$E^{\omega }\;X_{t}=x\left(t\right),\;E^{\omega }\;X_{t+\delta }=x\left(t+\delta \right).$$

The conditional expectation is

$$E^{\omega }\;\left(X_{t+\delta }|X_{t}\right)=\int_{\Omega }\xi \;\frac{\rho (\xi ,X_{t})}{\rho (X_{t})}d\xi ,$$

where $\xi \equiv X_{t+\delta }$ is used for notational convenience. However, by independence of the variables $\rho \left(X_{t+\delta },X_{t}\right)=\rho \left(X_{t+\delta }\right)\rho \left(X_{t}\right)$. Therefore, $E^{\omega }\;X_{t+\delta }=E^{\omega }\;\left(X_{t+\delta }|X_{t}\right)$. Since δ can be either positive or negative, the claim follows.  ☐

Consider the nonlinear problem, where the phase-space trajectory of a system is represented by a Hölder function x(t) (see Appendix A, Definition A2) and *t* is a real-valued parameter, for example time or curve length. Let us suppose that the continuous temporal evolution of a differential system can be represented by a generalization of the Langevin equation of the form

(1) $$dx\left(t\right)=a\left(x,t\right)dt+B\left(x,t\right)dt^{\beta },\;\beta <1,$$

where a(x,t) and *B* are bounded and measurable functions of the co-ordinates and furthermore a(x,t) is continuous in both *x* and *t*. That is, for all ϵ, such that 0≤ϵ≤dt

$$\Delta_{\epsilon }^{+}\left[x\right]\left(t\right)=x\left(t+\epsilon \right)-x\left(t\right)=a\left(x,t\right)\epsilon +B\left(x,t\right)\epsilon^{\beta }+O\left(\epsilon \right).$$

This can be recognized as the Hölder growth condition of order β, since a(x,t)ϵ is an $O\left(\epsilon \right)$ term. The fractional exponent β is treated as a free parameter with value to be determined later.

The type of admissible functions coupled to the fractional exponent depends critically on the assumption of continuity of the reconstructed trajectory. This in turn demands for the fluctuations of the fractional term to be discontinuous. The proof technique is introduced in [26], while the argument is similar to the one presented by Gillespie [8].

Without loss of generality, set a=0. Let $x_{t+\epsilon }=x_{t}+B\left(x_{t},t\right)+O\left(\epsilon^{\beta }\right)$ and $|\Delta_{\epsilon }x|\leq K\epsilon^{\beta }$. Fix the interval [t,t+ϵ] and choose a partition of points $P=\{t_{k}=t+\epsilon \;k/N\}$

$$x_{t_{k}}=x_{t_{k-1}}+B\left(x_{t_{k-1}},t_{k-1}\right){\left(\epsilon /N\right)}^{\beta }+O\left({\left(\epsilon /N\right)}^{\beta }\right).$$

Therefore, by induction

$$\Delta_{\epsilon }x=x_{t+\epsilon }-x_{t}=\frac{1}{N^{\beta }}\sum_{k=0}^{N-1}B\left(x_{t_{k}},t_{k}\right)\epsilon^{\beta }+O\left(N^{1-\beta }\epsilon^{\beta }\right).$$

If we suppose that *B* is continuous in *x*, implying also continuity in *t*, after taking supremum limit on both sides

$$\underset{\epsilon \to 0}{lim sup}\frac{\Delta_{\epsilon }x}{\epsilon^{\beta }}=N^{1-\beta }B\left(x_{t},t\right)=B\left(x_{t},t\right).$$

Therefore, either β=1 (which is forbidden by hypothesis) or else B=0 so that B(x,t) must oscillate from point to point if β<1. Then, let’s denote the set $\chi_{\beta }:=\left\{B\left(x_{t},t\right)\neq 0\right\}$.

The argument demonstrates that so-defined set is totally disconnected in the topology of the real line [26]. This allows for the choice of the algebra F, since we can demand that $\Omega \subseteq \chi_{\beta }$ has for elements the semi-open intervals $\left[\tau_{i},\tau_{j}\right),\;\tau_{i,j}\in \chi_{\beta }$. Furthermore, the initial system in Equation (1) is equivalent to the finite existence of the fractional velocity $B\left(x,t\right)=\upsilon_{+}^{\beta }x\left(\tau_{i}\right)\neq 0$, since the differential system can be recognized as fractional Taylor series [26]. In other words, the events in the probability space are the observations of non-vanishing values of the fractional velocity of the signal.

From now on, let $P_{\tau }\equiv P\subseteq F$. Without loss of generality, suppose that $O\left(N^{1-\beta }\epsilon^{\beta }\right)\leq 1$. The stochastic representation $x_{t}\mapsto \left(X_{t}\left(\omega \right),\rho \right)$ is such that

$$E\;\frac{\Delta_{\epsilon }X}{\epsilon^{\beta }}-O_{\epsilon }=\frac{1}{N^{\beta }}\sum_{k=0}^{N-1}E\;B\left(X_{t_{k}},t_{k}\right),\;\forall N.$$

Therefore, we demand that $B(X_{t},t)$ is F-measurable and $L^{\;2}\left(\Omega ,T\right)$ as a technical condition.

By the Hölder condition, $|x_{t_{k}}-x_{t_{k-1}}|\leq K_{k}\epsilon^{\beta }$ for some set of constants $K_{k}$. Then, by transfer,

$$|E\;B\left(X_{t_{k}},t_{k}\right)-E\;B\left(X_{t_{k-1}},t_{k-1}\right)|\leq K_{k}\epsilon^{\beta }.$$

Therefore, $E\;B(X_{t_{k}},t_{k})$ exists and is bounded. By the same argument,

$$E{\left(\Delta_{\epsilon }X\right)}^{2}\leq K_{k}^{k}\epsilon^{2\beta }.$$

Then, we proceed by induction. Let $K_{s}=\sup_{i}K_{i}^{2}$ from the above partition:

$${\left(\Delta_{\epsilon }x\right)}^{2}=\sum_{i,j=0}^{N-1}\Delta_{i}x\Delta_{j}x=\sum_{i=0}^{N-1}\Delta_{i}x^{2}+2\sum_{i<j}^{N-1}\Delta_{i}x\Delta_{j}x\leq 3K_{s}\;\epsilon^{2\beta }N^{1-2\beta }.$$

Therefore, for the embedded variable,

$$E{\left(\Delta_{\epsilon }X\right)}^{2}=E\sum_{i,j=0}^{N-1}\Delta_{i}X\Delta_{j}X=E\sum_{i=0}^{N-1}\Delta_{i}X^{2}+2E\sum_{i<j}^{N-1}\Delta_{i}X\Delta_{j}X\leq 3K_{s}\;\epsilon^{2\beta }N^{1-2\beta }.$$

Since $\Delta_{i}X\Delta_{j}X$ are independent by Lemma 1, $E\Delta_{i}X\Delta_{j}X=E\Delta_{i}XE\Delta_{j}X\leq K_{s}$. Therefore, $Var\left[\Delta_{\epsilon }X\right]\leq 3K_{s}\;\epsilon^{2\beta }N^{1-2\beta }$.

The argument can be specialized to β=1/2 where $Var\left[\Delta_{\epsilon }X\right]\leq K_{s}\;\epsilon^{2\beta }$. Therefore, the variance exists ∀N and the Central Limit Theorem holds. Since by Lemma 1 the process is Markovian, it must follow that in limit N→∞ the random process is Wiener.

Now suppose that a≠0. Then, since a(x) is continuous of bounded variation (BVC, see Appendix A), then a.e.,

$$Ea\left(X,t\right)=Ea\left(x+Z,t\right)=E\left(a\left(x,t\right)+a_{x}^{\prime }Z+O\left(Z\right)\right)=a\left(x,t\right),\;Z=X_{t}-x_{t}$$

and

$$Ea{\left(X,t\right)}^{2}=a{\left(x,t\right)}^{2}+{a_{x}^{\prime }}^{2}\sigma^{2},\;\sigma^{2}=EZ^{2},$$

with $\sigma^{2}$ existing by the previous argument. Therefore, $Var\left[\Delta_{\epsilon }X\right]\leq 3K_{s}\;\epsilon^{2\beta }N^{1-2\beta }-a{\left(x,t\right)}^{2}\epsilon^{2}\leq 3K_{s}\;\epsilon^{2\beta }N^{1-2\beta }$ by the same argument as in the previous case. Therefore, for β=1/2, the limit of the random process is Wiener.

Let us denote the limit Wiener process by $W_{t}$. Using the stationarity and self-similarity of the increments $\Delta_{\epsilon }^{+}W_{t}=\sqrt{\epsilon }\;N\left(0,1\right),$ where N(0,1) is a standard Gaussian random variable. Therefore, for β=1/2, the velocity can be regularized to a finite value if we take the expectation. That is,

$$\upsilon_{+}^{\beta }EW_{t}=0,$$

since $\Delta_{\epsilon }^{+}EW_{t}=0$. However,

$$\upsilon_{+}^{\beta }\;E\left|W_{t}\right|=\int_{0}^{\infty }\sqrt{\frac{2}{\pi }}e^{-z^{2}/2}dz=1\;.$$

The estimate holds a.s. since $P(W_{t}=0)=0$, where P denotes probability.

Finally, there is a function b(X,t), such that b(X,t)ξ=B(X,t), ξ∼N(0,1). This follows directly from the axiom of choice, since we can always choose ξ=1. Therefore, the last equation can be treated as a definition of b(X,t).

In summary, the following theorem can be formulated:

**Theorem** **1** (Gaussian stochastic embedding)**.** *Suppose that x(t) is β-differentiable of order β=1/2 in the interval T=[t,t+ϵ] and*

$$dx\left(t\right)=a\left(x,t\right)dt+B\left(x,t\right)dt^{\beta }$$

*for 0<dt≤ϵ, where a(x,t) is continuous in both x and t and $B\left(x,t\right)\in L^{\;2}\left(\Omega ,T\right)$ is bounded but discontinuous. Furthermore, let $\chi_{\beta }$ be the set of change (Definition A6) of f[T].*

*Then, x(t) can be embedded in a probability space (T⊗Ω,F,ρ), such that*

*1*. $\Omega \subseteq \chi_{\beta }$,
*2*. $X_{t}$ has i. i. d. Gaussian increments,
*3*. $EX_{t}=x\left(t\right)$ and
*4*. $\upsilon_{+}^{\beta }E\left(|X_{t}\left|\right|X_{t}=x\right)=\upsilon_{+}^{\beta }\left|x\left(t\right)\right|=\left|b\left(x,t\right)\right|$ hold almost sure.

*Furthermore, the stochastic differential equation*

$$dX_{t}=a\left(X_{t},t\right)dt+b\left(X_{t},t\right)dW_{t}$$

*holds a.s. In the last equation, $W_{t}$ is a standard Wiener process and*

b(X,t)ξ:=B(X,t),ξ∼N(0,1).

Such embedding can be also called a **consistent stochastic embedding**. This theorem allows for Nelson’s characterization of the Langevin diffusion process.

## 3. Nelson’s Characterization

The Langevin equation can describe equivalent quantum-mechanical systems in a path-wise manner. These are the so-called *conservative diffusions* of Carlen [27]. The existence of so-conceived QM particle paths was proven under certain reasonable conditions [27]. Starting from the generalized Langevin equation, the argument can be specialized to a Wiener driving process, which can be handled using the apparatus of Itô calculus. Consider the stochastic differential equation with continuous drift and diffusion coefficients

$$dX_{t}=a\left(X,t\right)dt+b\left(X,t\right)dW_{t},$$

where a(X,t) and b(X,t) are smooth functions of the co-ordinates and $dW_{t}$ are the increments of a Wiener process $dW_{dt}∼N\left(0,dt\right)$ adapted to the past filtration $F_{t>0}$ – i.e., starting from the initial state.

Let $EX_{t}=x\left(t\right)$. Following Nelson [2], the forward and backward and *drift*, respectively *diffusion* coefficients, can be identified as the averaged velocities [28]:

(2) $$\begin{array}{cc} a & =\lim_{dt\to 0}E\left(\left.\frac{X_{t+dt}-X_{t}}{dt}\right|X_{t}=x\right)=\frac{d}{dt}\left(x-b\sqrt{dt}\right), \end{array}$$

(3) $$\begin{array}{cc} \left|b\right| & =\lim_{dt\to 0}E\left(\left.\frac{\left|X_{t+dt}-X_{t}\right|}{\sqrt{dt}}\right|X_{t}=x\right)=\upsilon_{+}^{1/2}\left|x\right|. \end{array}$$

The evolution of the density of the process can be computed from the forward Fokker–Planck equation

(4) $$\begin{array}{c} \frac{\partial }{\partial t}\rho +\frac{\partial }{\partial x}\left(a\rho \right)-\frac{1}{2}\frac{\partial^{2}}{\partial x^{2}}\left(b^{2}\rho \right)=0, \end{array}$$

which can be recognized as a conservation law for the probability current *j*:

$$\frac{\partial }{\partial t}\rho +\frac{\partial }{\partial x}j=0,\;j:=a\rho -\frac{1}{2}\frac{\partial }{\partial x}b^{2}\rho .$$

Under the finite energy technical condition, there is a backwards process with the *same transition density*

$$dX_{t}=\hat{a}\left(X,t\right)dt+b\left(X,t\right)d{\hat{W}}_{t},$$

which is adapted to the future filtration $F_{t<T}$ – i.e., starting from the final state. This leads to the anticipative (i.e., anti-Itô or anticipative) stochastic integrals. This process has Fokker–Planck equation

(5) $$\begin{array}{c} \frac{\partial }{\partial t}\rho +\frac{\partial }{\partial x}\left(\hat{a}\rho \right)+\frac{1}{2}\frac{\partial^{2}}{\partial x^{2}}\left(b^{2}\rho \right)=0. \end{array}$$

Then, it follows that the Nelson’s *osmotic velocity* can be defined from

$$a-\hat{a}=b^{2}\frac{\partial }{\partial x}\log b^{2}\rho +\phi \left(t\right),$$

where ϕ(t) is an arbitrary $C^{\;1}$ function of time as $u:=\frac{1}{2}\left(a-\hat{a}\right)$ and the *current velocity* as

$$v:=\frac{1}{2}\left(a+\hat{a}\right)$$

so that a continuity equation holds for the density

$$\frac{\partial }{\partial t}\rho +\frac{\partial }{\partial x}v\rho =0.$$

Furthermore, Pavon [21] has established that the entropy production over the whole space is

$$H^{\prime }\left(t\right):=-\frac{d}{dt}\int_{R}\rho \log\rho \;dx^{3}=-\frac{2}{b^{2}}E\;uv.$$

Thus, for a Markov diffusion process,

$$E\frac{1}{b^{2}}\int_{s}^{r}uv\;dt=\frac{1}{2}\left(H(s)-H(r)\right)$$

for a constant *b*.

## 4. The Complex Velocity Operator in SR and SM Theories

Scale relativity treats velocity only as a difference quotient. This is a necessity due to the assumed non-differentiability of the trajectories. Non-differentiability leads to introduction of two velocity fields—forward and backward, depending on the direction of differentiation in time. These fields are assumed to be finite for small values of the time step *dt* but they diverge to infinity in the limit dt→0 in a standard analysis setting. Therefore, such velocity fields can be defined only up to a finite resolution underlying the physical phenomenon under study. The velocity fields are assumed to admit representation of the form of a sum of a “classical part” plus a correction of a resolution-dependent and diverging fractal part. The classical part corresponds to the **absolutely continuous** part of the trajectory, while the fractal part corresponds to the **singular** and possibly **oscillatory** parts. Since, at the level of physical description, there is no way to favor the forward rather than the backward velocity, the description should incorporate them on equal grounds, i.e., forming a bivariate vector field R⊗R

$$\begin{array}{c} v_{+}:=\frac{\Delta_{dt}^{+}x}{dt}\;⊗\;v_{+}:=\frac{\Delta_{dt}^{-}x}{dt}. \end{array}$$

This bivariate vector field is represented by a complex-valued vector field [29] as $v=V-iU\;\;\in R^{\;3}$ with components given by $U:=\frac{1}{2}\left(v_{+}+v_{-}\right),V:=\frac{1}{2}\left(v_{+}-v_{-}\right),$ where *V* is interpreted as the “classical” velocity and *U* is a new quasi-velocity quantity (i.e., the *osmotic* velocity in the terminology of Nelson). Under these assumptions, Nottale introduces a complexified material derivative, which is a pseudo-differential operator acting on scalar functions as

$$DF=\partial_{t}F+\left(v\cdot \nabla \right)F-i\sigma^{2}\;\nabla^{2}F,$$

where σ is a constant, quantifying the effect of changing the resolution scale.

Stochastic mechanics allows for a similar treatment of the complex of forward and backward diffusions. The drift, resp. diffusion coefficients can be further embedded in a complex space as proposed by Pavon [21]:

$$\begin{array}{cc} a\;⊗\hat{a} & \mapsto V:=v-iu,\\ X_{t+dt}\;⊗X_{t-dt} & \mapsto dX=\frac{1}{2}\left(X_{t+dt}+X_{t-dt}\right)-i\frac{1}{2}\left(X_{t+dt}-X_{t-dt}\right), \end{array}$$

so that the diffusion process becomes complex. It follows that

$$dX=Vdt+\frac{1-i}{2}b\;dW_{t}+\frac{1+i}{2}\hat{b}\;d{\hat{W}}_{t}.$$

In the case when $b=\hat{b},$

$$dX=Vdt+\frac{1-i}{2}b\left(dW_{t}+id{\hat{W}}_{t}\right)=Vdt+\frac{e^{-\frac{i\pi }{4}}}{\sqrt{2}}b\left(dW_{t}+id{\hat{W}}_{t}\right).$$

Therefore, we can designate a new complex stochastic variable

$$Z_{t}:=\frac{dW_{t}+id{\hat{W}}_{t}}{\sqrt{2}}.$$

Because of its double adaptation, $Z_{t}$ retains its local martingale properties: that is, $EZ_{t}=0$. In this case, notably $Var\;Z_{t}=0$, but $E|Z_{t}{|}^{2}=1$, so that finally,

$$dX=Vdt+\sqrt{-i}b\;dZ_{t}.$$

Therefore, a formal Itô differential can be introduced in exactly the same way

(6) $$dF=\frac{\partial }{\partial t}F+dX\frac{\partial }{\partial x}F+\frac{1}{2}\left[dX^{2}\right]\frac{\partial^{2}}{\partial x^{2}}F$$

with quadratic variation $\left[dX^{2}\right]=-ib^{2}dt$. Therefore, in components,

(7) $$dF=\left(\frac{\partial }{\partial t}F+V\frac{\partial F}{\partial x}-\frac{ib^{2}}{2}\frac{\partial^{2}}{\partial x^{2}}F\right)dt+\sqrt{-i}b\frac{\partial F}{\partial x}\;dZ_{t},$$

which generalize to

$$dF=\left(\partial_{t}F+\left(V\cdot \nabla \right)F-\frac{ib^{2}}{2}\nabla^{2}F\right)dt+\sqrt{-i}b\left(dZ_{t}\cdot \nabla \right)F$$

in three dimensions [28]. It is apparent that both theories share an identical algebraical structure, while SM can be considered as a stochastic representation of SR.

**Remark** **1.** Conceptually, the forward process can be interpreted as a prediction, while the backward process can be interpreted as a retrodiction.

Note that, in the complex formulation of Pavon, the real part of the driving process $Z_{t}$ corresponds to the forward (i.e., adapted to the past) process, while the imaginary part corresponds to the backward (i.e., adapted to the future) process. This is of course one of infinitely many choices, since the complex factor in the diffusion coefficient is a root of unity and hence represents a rotation in the complex plane.

*The martingale property of the complex Wiener process conceptually means that the knowledge of the past and future of the process do not bias the outcome at the present time (i.e., at measurement). Note that the mapping is invertible since*

$$X_{t+dt}=Re\left(dX\right)+Im\left(dX\right),\;|\;X_{t-dt}=Re\left(dX\right)-Im\left(dX\right).$$

*From these formulas, it is apparent that the real part, or respectively the imaginary part of the resulting process do not have separate meanings, as they mix the predictive process with the retrodictive process. To illustrate the point, suppose that $F=F_{r}+iF_{i}$ and $a=a_{r}+ia_{i}$ and the original process dX is transformed as F(X). Then, a straightforward calculation gives*

(8) $$\begin{array}{c} Re\left(dF\right)=\left(\frac{\partial F_{r}}{\partial t}+a_{r}\frac{\partial }{\partial x}F_{r}-a_{i}\frac{\partial }{\partial x}F_{i}+\frac{b^{2}}{2}\frac{\partial^{2}}{\partial x^{2}}F_{i}\right)dt\\ +\frac{b}{\sqrt{2}}\;\left(dW_{t}\left(\frac{\partial }{\partial x}F_{r}+\frac{\partial }{\partial x}F_{i}\right)+d{\hat{W}}_{t}\left(\frac{\partial }{\partial x}Fr-\frac{\partial }{\partial x}F_{i}\right)\right), \end{array}$$

(9) $$\begin{array}{c} Im\left(dF\right)=\left(\frac{\partial F_{i}}{\partial t}+a_{r}\frac{\partial }{\partial x}F_{r}+a_{i}\frac{\partial }{\partial x}F_{i}+\frac{b^{2}}{2}\frac{\partial^{2}}{\partial x^{2}}F_{r}\right)dt\\ -\frac{b}{\sqrt{2}}\;\left(dW_{t}\left(\frac{\partial }{\partial x}F_{r}-\frac{\partial }{\partial x}F_{i}\right)-d{\hat{W}}_{t}\left(\frac{\partial }{\partial x}Fr+\frac{\partial }{\partial x}F_{i}\right)\right). \end{array}$$

## 5. The Real Stochastic Geodesic Equations

The appearance of the Wiener process entails the application of the fundamental Itô Lemma for the forward (i.e., adapted to the past, plus sign) or the backward processes (i.e., adapted to the future, minus sign), respectively. In differential notation, it reads

(10) $$dF\left(X\right)=dX\frac{\partial }{\partial x}F\pm \frac{\left[dX^{2}\right]}{2}\frac{\partial^{2}}{\partial x^{2}}F,$$

where $\left[dX^{2}\right]=b^{2}dt$ is the quadratic variation of the process. It can be seen that in this case the (forward) differential operator *d* acts as a material derivative.

The term *geodesic* will be interpreted as a solution of a variational problem [30,31]. A brief treatment is given in Appendix B. By application of Itô’s Lemma, the forward geodesic equation can be obtained as:

(11) $$\frac{\partial }{\partial t}a+a\frac{\partial }{\partial x}a+\frac{b^{2}}{2}.\frac{\partial^{2}}{\partial x^{2}}a=0.$$

This can be recognized as a Burgers equation with negative kinematic viscosity for the drift field [13].

The backward geodesic equation follows from the application of the Itô’s lemma for the anticipative process

(12) $$\frac{\partial }{\partial t}a^{\prime }+a^{\prime }\frac{\partial }{\partial x}a^{\prime }-\frac{b^{2}}{2}\frac{\partial^{2}}{\partial x^{2}}a^{\prime }=0$$

This can be recognized as a Burgers equation with positive kinematic viscosity for the drift field.

The solution of the Burgers equation is well known and can be given by the convolution integrals (Equation (44)) for the case of positive viscosity [13]. The case about the negative viscosity can not be easily solved using Fourier transform. Therefore, a different solution technique will be pursued. Time-varying solutions will be constructed from topological deformations of the stationary solutions.

In QM applications, b=ℏ/2m. Normalization b=1 will be assumed further in most cases to simplify calculations.

### 5.1. Path-Wise Separable Solutions

In the first instance, one can solve the geodesic equation by supposing separability. By making the ansatz a(x,t)=f(x)g(t), we arrive at the equation:

$$\frac{f^{\prime \prime }\left(x\right)}{2f(x)}+g\left(t\right)\;f^{\prime }\left(x\right)+\frac{g^{\prime }\left(t\right)}{g(t)}=0.$$

This has the unique solution

(13) $$a\left(x,t\right)=\frac{x+x_{0}}{t+T}.$$

The resulting Itô equation can be formulated as

$$dX=\frac{X+x_{0}}{t+T}dt+dW_{t}.$$

The stochastic differential equation for the drift is therefore

$$da=\frac{1}{t+T}dW_{t},$$

which can be integrated exactly in Itô’s sense as

(14) $$a\left(t\right)=a_{0}+\int_{0}^{t}\frac{dW_{s}}{s+T},\;a_{0}=\frac{x_{0}}{T}.$$

**Figure 1:**
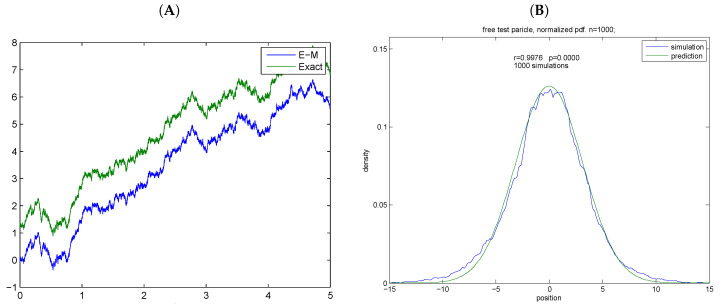
Virtual trajectories of the separable process. (**A**) virtual trajectories; (**B**) empirical vs. theoretical density. (**A**) exact simulation of separable process is compared with the Euler–Maruyama algorithm. E—Exact simulation, E–M—Euler–Maruyama simulation; An offset is added to the exact solution for appreciation. Time is given in arbitrary units; (**B**) the empirical transition density is estimated from $n=\log^{2}\left(N_{s}\;N\right)$ bins. Pearson’s correlation is given as an inset—r = 0.9976.

Therefore,

(15) $$X\left(t\right)=\frac{x_{0}}{T}\left(t+T\right)+\left(t+T\right)\int_{0}^{t}\frac{dW_{s}}{T+s},$$

where *T* is the stopping time. Therefore, an exact numerical quadrature can be performed (Figure 1)

The corresponding density can be obtained from the Fokker–Planck equation

$$\frac{\partial }{\partial t}\rho +\frac{\partial }{\partial x}\left(\frac{\rho \;x}{t+T}\right)-\frac{1}{2}\frac{\partial^{2}}{\partial x^{2}}\rho =0$$

with solution

(16) $$\rho \left(x,t\right)=\frac{1}{\sqrt{2\pi \left(T+t\right)}}\exp\left(\frac{x^{2}}{2(t+T)}\right).$$

It should be noted that, under time reversion, we arrive at the same solution, which however leads to a different Fokker–Planck equation

$$\frac{\partial }{\partial t}\rho +\frac{\partial }{\partial x}\left(\frac{\rho \;x}{t-T}\right)+\frac{1}{2}\frac{\partial^{2}}{\partial x^{2}}\rho =0$$

with solution

$$\rho \left(x,t\right)=\frac{1}{\sqrt{2\pi \left(T-t\right)}}\exp\left(-\frac{x^{2}}{2(t-T)}\right),$$

which can be recognized as a Brownian bridge. The entropy of this density can be calculated as

$$H\left(t\right)=\frac{\log2\pi \left(t-T\right)+1}{2}.$$

### 5.2. Stationary Drift Fields

For time-homogeneous diffusion, the geodesic equation can be brought into the form

$$\frac{1}{2}\frac{\partial }{\partial x}\left(a^{2}+\frac{\partial }{\partial x}a\right)=0,$$

which can be integrated once to give

$$a^{2}+\frac{\partial }{\partial x}a=-E.$$

The integration constant *E* can be identified with the energy. The resulting first order ordinary differential equation (ODE) can be solved as

(17) $$\begin{array}{cc} a(x) & =-\sqrt{E}\;\tan\left(\sqrt{E}\;x+c\right),\;E>0, \end{array}$$

(18) $$\begin{array}{cc} a(x) & =\frac{1}{x+c},\;E=0. \end{array}$$

The solution for E>0 was identified by Herman [32]. By translation, invariance of the coordinates, c=0 is admissible. This observation will be used further for the transient solution. The link between the two solutions can be established as follows. Note that

$$a\left(x\right)=\sqrt{E}\;\cot\sqrt{E}\;x$$

is also a solution. Then,

$$\lim_{E\to 0}\sqrt{E}\;\cot\sqrt{E}\;x=\frac{1}{x},$$

which is the second solution.

The expectation of the trajectory can be obtained by solving the ODE

$$\frac{dx}{dt}=-\sqrt{E}\;\tan\sqrt{E}\;x$$

so that

(19) $$\begin{array}{cc} \sqrt{E}\;x\left(t\right) & =\arcsin e^{-Et+c}, \end{array}$$

(20) $$\begin{array}{cc} \sqrt{E}\;x\left(t\right) & =\arccos e^{-Et+c}. \end{array}$$

In accordance with so-developed theory for c=0,

$$\upsilon_{+}^{1/2}x\left(t=0\right)=\pm \lim_{h\to 0+}E\frac{2\sqrt{h}\;e^{-Eh}}{\sqrt{1-e^{-2Eh}}}=\pm \sqrt{2E}.$$

Furthermore, for E=0,

$$\frac{dx}{dt}=\pm \frac{1}{x}$$

so that in the same way

$$x\left(t\right)=c\pm \sqrt{2t}.$$

The backward geodesic equation

$$\frac{1}{2}\frac{\partial }{\partial x}\left(a^{2}-\frac{\partial }{\partial x}a\right)=0$$

by the same method leads to

(21) $$\begin{array}{cc} a(x) & =-\sqrt{E}\;\tanh\left(\sqrt{E}\;x+c\right),\;E>0, \end{array}$$

(22) $$\begin{array}{cc} a(x) & =-\frac{1}{x+c},\;E=0. \end{array}$$

### 5.3. Stationary Density Solutions

The stationary density ρ(y) is a solution of the Fokker–Planck (i.e., forward Kolmogorov) equations parametrized by *E*:

(23) $$\begin{array}{cc} \frac{1}{2}\frac{\partial^{2}}{\partial y^{2}}\rho {\left(y^{2}+1\right)}^{2} & =0,\;E>0, \end{array}$$

(24) $$\begin{array}{cc} \frac{1}{2}\frac{\partial^{2}}{\partial y^{2}}\rho \;y^{4} & =0,\;E=0. \end{array}$$

The case E>0 leads to

(25) $$\begin{array}{c} \frac{\partial }{\partial x}\tan\left(x\right)\rho +\frac{1}{2}\frac{\partial^{2}}{\partial x^{2}}\rho =0 \end{array}$$

with stationary solution

$$\rho =\cos^{2}\sqrt{E}x,$$

which can be valid on a bounded domain. The entropy of this solution in the domain $\left[-\pi /\left(2\sqrt{E}\right),\pi /\left(2\sqrt{E}\right)\right]$ can be calculated as

$$H=\frac{\pi \log4}{2}-\frac{\pi }{2}.$$

The case E=0 leads to

(26) $$\begin{array}{c} \frac{\partial }{\partial x}\frac{\rho }{x}-\frac{1}{2}\frac{\partial^{2}}{\partial x^{2}}\rho =0 \end{array}$$

with a stationary solution

ρ=|x|,

which can be valid on a bounded domain.

### 5.4. Transient Drift Fields

The solution of the Burgers equation is well known and can be given by the convolution integrals (Equation (44)) for the case of positive viscosity [13]. The case of negative viscosity emerging here is more challenging and it will be solved by a deformation of the stationary solution, so that in limit the stationary solution is recovered:

$$\lim_{t\to \infty }a\left(t,x\right)=a\left(x\right),\;E>0.$$

The solution is sought in the form (neglecting scale factors)

$$a\left(t,x\right)=-\frac{\sin x}{\cos x+f(t),}$$

which results in a linear ODE for the unknown function f(t):

$$\frac{2\;f^{\prime }\left(t\right)+f\left(t\right)}{2{\left(\cos x+f(t)\right)}^{2}}\;\sin x=0.$$

**Figure 2:**
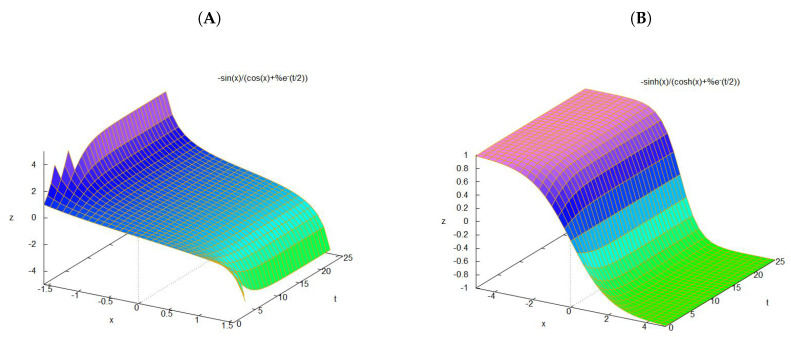
Time-varying drift fields for E=1, k=1. (**A**) forward drift; (**B**) backward drift.

By variation of the parameters, the solution for a(t,x) is given as

(27) $$a\left(t,x\right)=-\sqrt{E}\frac{\sin\sqrt{E}x}{\cos\sqrt{E}x+ke^{-\frac{Et}{2}}},$$

where the constant *E* represents an energy scale and *k* is an arbitrary constant. We can assume normalization, for example $k=\pm \sqrt{E}$, such that $a(t,\frac{\pi }{2E})=\pm 1$. Plots are presented in Figure 2.

The transformed Itô drift equation for k=1 reads

$$da\left(t,x\right)=-E\frac{e^{-Et/2}\cos\sqrt{E}x+1}{{\left(e^{-Et/2}+\cos\sqrt{E}x\right)}^{2}}dW_{t}=-\sqrt{E}\frac{e^{-Et/2}\cos\sqrt{E}x+1}{{\left(e^{-Et/2}+\cos\sqrt{E}x\right)}^{2}}dW_{E\;t}.$$

It can be further noticed that rescaling in a pair of new variables $x^{\prime }=\sqrt{E}x,\;t^{\prime }=Et$ leaves the ratio

$$z=\frac{x^{2}}{t}=\frac{x^{\prime 2}}{t^{\prime }}$$

invariant so that *z* becomes a similarity variable.

Furthermore, a formal forward Kolmogorov equation can be written in the y=a(t,x) variable with E=1 as

$$\frac{\partial }{\partial t}\rho -\frac{1}{2}\frac{\partial^{2}}{\partial y^{2}}\frac{\rho e^{t}\;{\left(\cos\left(x\right)+e^{\frac{t}{2}}\right)}^{2}}{{\left(e^{\frac{t}{2}}\;\cos\left(x\right)+1\right)}^{4}}=0,\;x=\pm \arcsin\left(\frac{e^{-\frac{t}{2}}y\;\left(\sqrt{\left(e^{t}-1\right)\;y^{2}+e^{t}}-1\right)}{y^{2}+1}\right)\;,$$

however its solution is challenging due to its mixed nonlinearity and will not be attempted here. Nevertheless, the analysis presented so far assures that asymptotically ρ can be obtained as a solution of the stationary equation.

The backward geodesic equation leads to the following solution :

(28) $$a{\left(t,x\right)}^{\prime }=-\sqrt{E}\frac{\sinh\sqrt{E}x}{\cosh\sqrt{E}x+ke^{-\frac{Et}{2}}}.$$

### 5.5. Asymptotic Density Solutions

The forward drift itself a≡y (symbol changed) obeys the transformed stochastic differential equations

(29) $$\begin{array}{cc} E>0: & \;dy=\sqrt{E}\;\left(y^{2}+1\right)\;dW_{t}=\left(y^{2}+1\right)\;dW_{E\;t}, \end{array}$$

(30) $$\begin{array}{cc} E=0: & \;dy=y^{2}\;dW_{t}. \end{array}$$

The density ρ is a solution of the forward Kolmogorov equations parametrized by *E*:

(31) $$\begin{array}{cc} \frac{\partial }{\partial E\;t}\rho & =\frac{1}{2}\frac{\partial^{2}}{\partial y^{2}}\rho {\left(y^{2}+1\right)}^{2},\;E\neq 0, \end{array}$$

(32) $$\begin{array}{cc} \frac{\partial }{\partial t}\rho & =\frac{1}{2}\frac{\partial^{2}}{\partial y^{2}}\rho \;y^{4},\;E=0. \end{array}$$

The solutions can be obtained using the Laplace transform $L_{s}f\left(t\right)\mapsto \hat{f}\left(s\right)$. In this way, the partial differential equation can be transformed into an ODE for the Laplace variable:

(33) $$\begin{array}{c} -\frac{1}{2}\frac{\partial^{2}}{\partial y^{2}}\hat{\rho }y^{4}+\hat{\rho }\;s=\rho \left(0,y\right), \end{array}$$

(34) $$\begin{array}{c} -\frac{1}{2}\frac{\partial^{2}}{\partial y^{2}}\hat{\rho }{\left(y^{2}+1\right)}^{2}+\hat{\rho }\;s=\rho \left(0,y\right). \end{array}$$

To obtain the Green’s function, we take homogeneous initial conditions a.e. The solutions in the time domain can be obtained by the inverse Laplace transformation:

(35) $$\begin{array}{cc} \hat{\rho }\left(s,y\right) & =\frac{Ae^{-\frac{\sqrt{2\;s}}{y}}}{\sqrt{s}\;y^{3}}\overset{L_{s}^{-1}}{\to }\rho \left(t,y\right)=\frac{A}{\sqrt{t}\;y^{3}}e^{-\frac{1}{2\;t\;y^{2}}}, \end{array}$$

(36) $$\begin{array}{cc} \hat{\rho }\left(s,y\right) & =\frac{e^{-\sqrt{2s/E-1}\;\arctan\left(y\right)}}{{\left(y^{2}+1\right)}^{\frac{3}{2}}}\overset{L_{s}^{-1}}{\to }\rho \left(t,y\right)=\frac{e^{-\frac{\arctan^{2}y-{\left(Et\right)}^{2}}{2E\;t}}}{\sqrt{\pi \;Et}\;{\sqrt{\left(y^{2}+1\right)}}^{3}}. \end{array}$$

In the position space, the solution can be obtained using Grisanov’s theorem [4]:

(37) $$\begin{array}{cc} \rho (t,x) & =\frac{\left|x\right|}{\sqrt{t}}\exp\left(-\frac{x^{2}}{2\;t}\right), \end{array}$$

(38) $$\begin{array}{cc} \rho (t,x) & =\frac{|\cos\left(\sqrt{E}\;x\right)|}{\sqrt{\pi E\;t}}\exp\left(\frac{Et}{2}-\frac{x^{2}}{2t}\right). \end{array}$$

The second equation is not acceptable from a physical point of view since $\lim_{t\to \infty }\rho \left(t,x\right)$ diverges.

In the same way for the backward drift,

(39) $$\begin{array}{cc} E>0: & \;dy=\sqrt{E}\;\left(y^{2}-1\right)\;dW_{t}=\left(y^{2}-1\right)\;dW_{E\;t}, \end{array}$$

(40) $$\begin{array}{cc} E=0: & \;dy=y^{2}\;dW_{t}, \end{array}$$

(41) $$\begin{array}{cc} \frac{\partial }{\partial E\;t}\rho & =\frac{1}{2}\frac{\partial^{2}}{\partial y^{2}}\rho {\left(y^{2}-1\right)}^{2},\;E\neq 0, \end{array}$$

with solutions

$$\rho \left(t,y\right)=\frac{1}{\sqrt{\pi \;Et}\;{\sqrt{\left(y^{2}-1\right)}}^{3}}\exp\left(-\frac{{\mathrm{arctanh}}^{2}y+{\left(Et\right)}^{2}}{2E\;t}\right)$$

in the drift space and in position space

$$\rho \left(t,x\right)=\frac{\cosh\left(\sqrt{E}\;x\right)}{\sqrt{\pi E\;t}}\exp\left(-\frac{x^{2}}{2t}-\frac{Et}{2}\right),$$

respectively. This is acceptable from a physical point of view since $\lim_{t\to \infty }\rho \left(t,x\right)=0$, which is a correct asymptotic behavior.

## 6. The Complex Stochastic Geodesic Equations

The complexification removes the restriction of positive definiteness of the *E* parameter so that the substitution t↦±Et becomes admissible by an appropriate cut along the complex plane.

In a similar way, for the complex case, we have

$$dX_{t}=-i\sqrt{E}\tanh\sqrt{E}X_{t}\;dt+\sqrt{-i}\;dZ_{t},$$

which, under substitution, y=tanhx leads to

$$dy=\sqrt{-i}\sqrt{E}\;\left(y^{2}-1\right)\;dZ_{t},$$

By the same methods as used above, the asymptotic density for the drift variable can be obtained as

$$\rho \left(t,y\right)=Re\frac{1}{\sqrt{\pi t}\;{\left(y^{2}-1\right)}^{\frac{3}{2}}}\exp\left(\frac{iEt}{2}-\frac{i{\mathrm{arctanh}}^{2}y}{2Et}\right).$$

For the resulting density in the position space, it can be calculated that

(42) $$\begin{array}{c} \rho \left(t,x\right)=Re\frac{i\;\cosh\left(\sqrt{E}x\right)}{\sqrt{\pi Et}}\;\exp\left(\frac{iEt}{2}-\frac{ix^{2}}{2t}\right).\; \end{array}$$

In a similar way, for the other solution,

$$dX=i\sqrt{E}\tan\sqrt{E}Xdt+\sqrt{-i}\;dZ_{t},$$

which under substitution $y=\tan\sqrt{E}x$ leads to the drift equation

$$dy=\sqrt{-i}\sqrt{E}\left(1+y^{2}\right)\;dZ_{t}.$$

The drift density can be readily obtained as

$$\rho \left(t,y\right)=\frac{1}{\sqrt{\pi t}\;{\left(y^{2}+1\right)}^{\frac{3}{2}}}\exp\left(-\frac{iEt}{2}-\frac{i\arctan^{2}y}{2Et}\right).$$

In the position space, the density is of the form

(43) $$\begin{array}{c} \rho \left(t,x\right)=Re\frac{|\cos\left(\sqrt{E}x\right)|}{\sqrt{\pi Et}}\exp\left(-\frac{ix^{2}}{2t}-\frac{iEt}{2}\right). \end{array}$$

In either case, the densities asymptotically approach zero.

## 7. Real-Valued and Complex Cole–Hopf Transformations

The Burgers equation can be linearized by the Cole–Hopf transformation [33,34]. This mapping transforms the nonlinear Burgers equation into the linear heat conduction equation in the following way. Let

$$u=\frac{\partial }{\partial x}\log a.$$

Substitution into Equation (11) leads to

$$\frac{1}{2\;u^{2}}\left(u\;\frac{\partial^{3}u}{\partial x^{3}}+2u\;\frac{\partial^{2}u}{\partial t\partial x}-\frac{\partial u}{\partial x}\;\frac{\partial^{2}u}{\partial x^{2}}-2\frac{\partial u}{\partial t}\;\frac{\partial u}{\partial x}\right)=0.$$

This can be recognized as

$$\frac{\partial }{\partial x}\frac{1}{u}\left(\frac{\partial }{\partial t}u+\frac{1}{2}\frac{\partial^{2}}{\partial x^{2}}u\right)=0,$$

which is equivalent to a solution of the equation

$$\frac{\partial }{\partial t}u+\frac{1}{2}\frac{\partial^{2}}{\partial x^{2}}u=0.$$

It should be noted that if instead of the forward development (i.e., prediction) one takes the backward development (i.e., retrodiction), the usual form of the Burgers equation is recovered. This corresponds to the anticipative Wiener process, which is subject to the anticipative Itô calculus [17,35]:

$$\frac{\partial }{\partial t}\hat{a}+\hat{a}\frac{\partial }{\partial x}\hat{a}-\frac{1}{2}\frac{\partial^{2}}{\partial x^{2}}\hat{a}=0.$$

In this case, the usual general solution can be revealed

(44) $$\begin{array}{cc} \phi_{0}\left(x\right) & =\exp\left(\frac{1}{2\nu }\int_{0}^{x}{\hat{a}}_{0}\left(u\right)du\right), \end{array}$$

(45) $$\begin{array}{cc} \hat{a}\left(x,t\right) & =\frac{\partial }{\partial x}\log\frac{1}{2\sqrt{\pi \nu t}}\int_{-\infty }^{\infty }\phi_{0}\left(u\right)e^{-\frac{{\left(x-u\right)}^{2}}{4\nu t}}du=\frac{\int_{-\infty }^{\infty }\frac{x-u}{t}\phi_{0}\left(u\right)e^{-\frac{{\left(x-u\right)}^{2}}{4\nu t}}du}{\int_{-\infty }^{\infty }\phi_{0}\left(u\right)e^{-\frac{{\left(x-u\right)}^{2}}{4\nu t}}du}, \end{array}$$

where ν=1/2 is the viscosity coefficient.

In the complex case, starting from the generalized Itô differential, the complex velocity field becomes

$$dV=\left(\frac{\partial }{\partial t}V+V\frac{\partial }{\partial x}V-\frac{ib^{2}}{2}\frac{\partial^{2}}{\partial x^{2}}V\right)dt+\sqrt{-i}b\frac{\partial }{\partial x}V\;dZ_{t}.$$

The geodesic equation reads

EdV=0.

Therefore, by the martingale property, this is equivalent to

$$\frac{\partial }{\partial t}V+V\frac{\partial }{\partial x}V-\frac{ib^{2}}{2}\frac{\partial^{2}}{\partial x^{2}}V=0,$$

which can be recognized as a generalized Burgers equation with imaginary kinematic viscosity coefficient. Applying the complex Cole–Hopf transformation as [36]

$$V=-i\frac{\partial }{\partial x}\log U,\;-\pi <\arg U<\pi$$

and specializing to b=1 leads to

$$-\frac{U\;\left(\frac{\partial^{3}}{\partial x^{3}}U\right)-\left(\frac{\partial }{\partial x}U\right)\;\left(\frac{\partial^{2}}{\partial x^{2}}U\right)-2i\left(\frac{\partial }{\partial t}U\right)\;\left(\frac{\partial }{\partial x}U\right)+2iU\;\left(\frac{\partial^{2}}{\partial t\partial x}U\right)}{2U^{2}}=0,$$

which can be recognized as a gradient

$$-\frac{\partial }{\partial x}\frac{1}{U}\left(i\frac{\partial }{\partial t}U+\frac{1}{2}\frac{\partial^{2}}{\partial x^{2}}U\right)=0.$$

The last equation is equivalent to the solution of the free Schrödinger equation. On the other hand, the diffusion part is simply

$$-\sqrt{i}\left(\frac{\partial^{2}}{\partial x^{2}}\log U\right)dZ_{t}=-\sqrt{i}\left(\frac{\partial }{\partial x}\frac{1}{U}\frac{\partial }{\partial x}U\right)$$

since $-i\sqrt{-i}=-\sqrt{i}$.

This corresponds with the arguments given in [37] that the coefficient of the stochastic noise should be purely imaginary. Calculations can be reproduced in the computer algebra system Maxima [38].

## 8. Numerical Results

The different types of solutions of the stochastic geodesic equation were simulated using the Euler–Maruyama algorithm. Simulations were performed in Matlab. An example of a simulation script is given in Appendix C.

### 8.1. Exact Simulations

The exact simulations of the separable process were compared to simulations computed by the Euler–Maruyama algorithm. Achieved correlation was 1.0 while the mean squared error was on the order of $1\times 10^{-8}$.The comparison is presented in Figure 1. The empirical transition density was computed from $N_{s}=1000$ simulations and correlated to the graph of Equation (16). The theoretical density was computed from Equation (16) for stopping time T=10. Achieved correlation was 0.9976.

### 8.2. Free Diffusion

**Figure 3:**
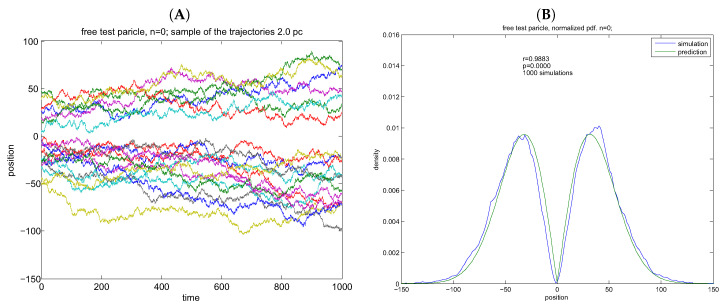
Simulations of free particles virtual trajectories. (**A**) virtual trajectories: free particles; (**B**) empirical vs. theoretical density. Simulations are based on N=10,000 points in $N_{s}=1000$ simulations. (**A**,**B**) width of potential well is 2L=100 units. The empirical *pdf* is estimated from $n=\log^{2}\left(N_{s}\;N\right)$ bins. Pearson’s correlations are given as inset—r = 0.9883. Norming of the free particle transient results in Rayleigh density.

The normalized asymptotic transient density of the free particle distribution can be recognized as the Rayleigh’s distribution (Figure 3)

$$R\left(x,t\right)=\frac{\left|x\right|}{2t}e^{-\frac{x^{2}}{2\;t}}.$$

### 8.3. Particle in a Box

The third simulated case comprised a freely diffusing particle in a square potential well of size 2L. The approach was based on Hermann [32]. Individual trajectories were simulated according to the fundamental equation using the scheme of Euler–Maruyama:

$$x_{n+1}=x_{n}-2D\Delta t\frac{\pi n}{L}\tan\left(\frac{\pi n}{L}x_{n}-\pi \frac{n+1}{2}\right)+\sqrt{2D\Delta t}\;\Delta W_{n},$$

where $\Delta W_{n}∼N\left(0,1\right)$.

Restarting boundary conditions were used for the simulations to avoid distortions of the distribution. That is, if a simulated particle crossed the boundaries its position was reset to its original position.

The initial particle positions were sampled from a uniform distribution between −L and *L*. The theoretical density for the particle in a box case is given by

$$\rho_{s}\left(x\right)=\frac{2}{L}\;\sin^{2}\left(n\pi \left(\frac{x}{L}+\frac{1}{2}\right)\right).$$

Results are based on N=10000 points in $N_{s}=1000$ simulations.

**Figure 4:**
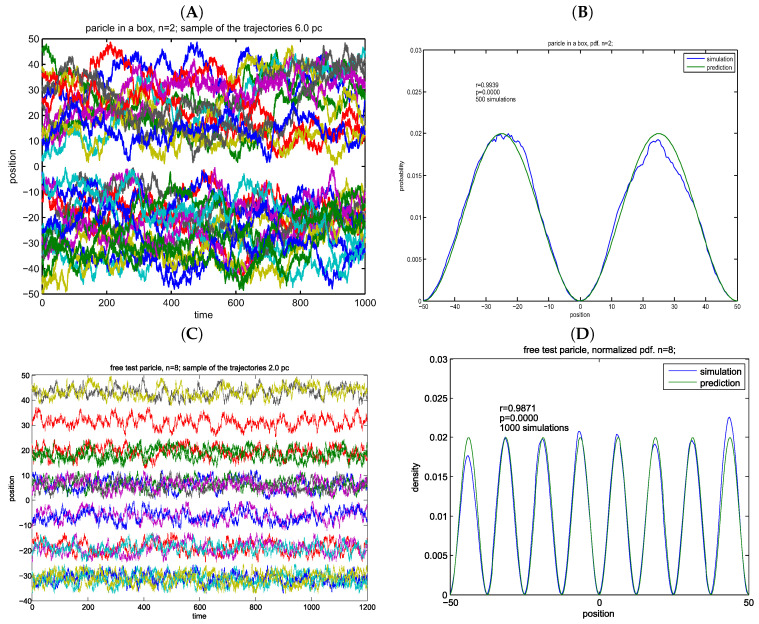
Simulations of particles in a box for two quantum numbers. (**A**) virtual trajectories: particle in a box, n = 2; (**B**) empirical vs. theoretical density; (**C**) test particles in a box, n = 8; (**D**) empirical vs. theoretical density. (**A**,**B**) width of potential well is 2L=100 units.

The empirical *pdf* is estimated from $n=\log{\left(N_{s}\;N\right)}^{2}$ bins. Pearson’s correlations are given as insets: B – r = 0.9939, D – r = 0.9871. For both cases, the numerical precision correlates excellently with the analytical solutions (Figure 4).

## 9. Discussion

This work was motivated in part by the premise that inherently nonlinear phenomena need development of novel mathematical tools for their description. The relaxation of the differentiability assumption opens new avenues in describing physical phenomena, as demonstrated by SM and SR, but also challenges existing mathematical methods, which are developed for smooth signals [2,3]. While this description can be achieved also by fractional differ–integrals, or by multi-scale approaches [39], the present work focused on a local description. The reason for this choice is that locality provides a direct way of physical interpretation of the obtained results. In this regard, Hölderian functions can be used as building blocks of such strongly nonlinear models, which give rise to singular [24,40] or non-differentiable models.

The second motivation of the present work was to investigate the potential of stochastic methods for simulations of quantum-mechanical and convection-diffusive systems. While the usual presentation of the stochastic mechanics typically used the Schrödinger equation as a solution device and paths were constructed from solutions of the Schrödinger equation, this is not necessary. McClendon and Rabitz simulated several quantum systems using the differential equations of Nelson’s stochastic quantization as a starting point [41]. In the framework of scale relativity, Herman [32] and later Al Rashid et al. [42] simulated QM particle in a box using the Langevin equations. Later, Al-Rashid et al. [43] simulated the quantum harmonic oscillator extending Herman’s approach. The approach presented here can be used as an alternative to numerical solutions of the Schrödinger equation. In this scenario, the density of the solution can be sampled from Monte Carlo simulations as demonstrated. Presented numerical approaches can be used, for example, for simulations of nanoparticles or quantum dots, which are mesoscopic objects and are expected to have properties intermediate between macroscopic and quantum systems [44]. This can be of interest, for example in sedimentation studies, where Langevin dynamics was proposed [45]. In principle, presented results can be extended towards asynchronous simulations using the Gillespie’s algorithm [8]. This can be achieved using time steps distributed exponentially.

Obtained results can be also discussed in view of the fluctuation-dissipation relationships. The fluctuation-dissipation theorem relates the linear response relaxation of a system from a non-equilibrium state to the properties of fluctuations in equilibrium. This is an exact result in the case of the Ornstein–Uhlembeck process, where the drift term is linear. The geodesic treatment in the present work provides a different relationship between the drift and a(x,t) and diffusivity b(x,t). In the small perturbation regime around the equilibrium the geodesic process $x_{eq}\left(t\right)$ can be approximated by an Ornstein–Uhlembeck process for the fluctuation term (ξ=δx(t)), therefore an appropriate fluctuation-dissipation theorem can be formulated assuming that equipartition also holds.

A fact that is not fully addressed by both stochastic mechanics and scale relativity is why do the theories work only for (box) fractal dimension 2 of the paths. While Nottale gives an heuristic argument and claims that the prescription of a Wiener process may be generalized, he does not proceed to rigorously develop the argument. On the other hand, the stochastic mechanics fixes from the start the Wiener process as a driving noise. While this may look plausible in view of the traditions in the treatment of Brownian motion, it is a choice that should be justified as nowadays anomalous types of diffusion dynamics are also recognized and systematically investigated (overview in [46]). The answer to this question can be given more easily by an approach inspired by Nottale and is partially given by the argument given by Gillepsie [8]. The original argument in [8] contains an explicit assumption of existence of the second moment of the distribution, which amounts to assuming Hölder continuity of order 1/2 as demonstrated here in Theorem 1. The theorem also corresponds to the result established for fractal interpolation computed via a chaos game where the limit random distribution has been identified with the Gaussian distribution [47,48].

While in SR particle ’trajectories’ are considered to be only virtual, SM and the original formulation of Bohm’s quantum mechanics treat them as physically real. It is noteworthy that recently Flack and Hiley [49] demonstrated that that a Bohm ’trajectory’ is the average of an ensemble of actual individual stochastic Feynman paths. This is in line with the treatment of the problem by the stochastic mechanics and scale relativity and promotes the view that Bohm’s quantum mechanics is a mean field theory of the stochastic mechanics.

## Appendix A. Notations, General Definitions and Properties of Fractional Velocity

**Definition** **A1** **(Asymptotic** **O** **notation)** *The notation $O\left(x^{\alpha }\right)$ is interpreted as the convention that*

$$\lim_{x\to 0}\frac{O\left(x^{\alpha }\right)}{x^{\alpha }}=0$$

*for α>0. The notation $O_{x}$ will be interpreted to indicate a Cauchy-null sequence with no particular power dependence of x.*

**Definition** **A2.** We say that f is of (point-wise) Hölder class $H^{\;\beta }$ if for a given x there exist two positive constants C,δ∈R that for an arbitrary y∈Dom[f] and given |x−y|≤δ fulfill the inequality $\left|f\left(x\right)-f\left(y\right)\right|\leq {C|x-y|}^{\beta }$, where |·| denotes the norm of the argument.

**Definition** **A3.** *Define the parametrized difference operators acting on a function f(x) as*

$$\begin{array}{cc} \Delta_{\epsilon }^{\pm }\left[f\right]\left(x\right) & :=sgn\left(\epsilon \right)\left(f(x+\epsilon )-f(x)\right). \end{array}$$

*The first one we refer to as forward difference operator, the second one we refer to as backward difference operator.*

**Definition** **A4.** *Define Fractional Variation operators of order 0≤β≤1 as*

(A1) $$\begin{array}{c} \upsilon_{\epsilon \pm }^{\beta }\left[f\right]\left(x\right):=\frac{\Delta_{\epsilon }^{\pm }\left[f\right]\left(x\right)}{{\left|\epsilon \right|}^{\beta }}. \end{array}$$

This section follows the presentation given recently in [50].

**Definition** **A5** **(Fractional** **order** **velocity)** *Define the* *fractional velocity* *of fractional order β as the limit*

(A2) $$\begin{array}{cc} \upsilon_{\pm }^{\beta }f\left(x\right) & :=\lim_{\epsilon \to 0}\frac{\Delta_{\epsilon }^{\pm }\left[f\right]\left(x\right)}{{\left|\epsilon \right|}^{\beta }}, \end{array}$$

*where 0<β≤1 are real parameters and f(x) is real-valued function. A function for which at least one of $\upsilon_{\pm }^{\beta }f\left(x\right)$ exists finitely will be called β-differentiable at the point x.*

In the above definition, we do not require upfront equality of left and right β-velocities. This amounts to not demanding continuity of the β-velocities in advance. Instead, continuity is a property, which is fulfilled under certain conditions.

**Definition** **A6.** *The set of points where the fractional velocity exists finitely and $\upsilon_{\pm }^{\beta }f\left(x\right)\neq 0$ will be denoted as the* ***set of change*** *$\chi_{\pm }^{\beta }\left(f\right):=\left\{x:\upsilon_{\pm }^{\beta }f\left(x\right)\neq 0\right\}$.*

Since the set of change $\chi_{+}^{\alpha }\left(f\right)$ is totally disconnected [26], some of the useful properties of ordinary derivatives, notably the continuity and the semi-group composition property, are lost.

**Definition** **A7.** *β-Regularized derivative of a function is defined as:*

$$\frac{d{\;}^{\beta \pm }}{dx}f\left(x\right):=\lim_{\epsilon \to 0}\frac{\Delta_{\epsilon }^{\pm }\left[f\right]\left(x\right)-\upsilon_{+}^{\beta }f\left(x\right)\epsilon^{\beta }}{\epsilon }.$$

*We will require as usual that the forward and backward regularized derivatives be equal for a uniformly continuous function.*

In this section, we assume that the functions are BVC in the neighborhood of the point of interest. Under this assumption, we have

- Product rule
  $$\begin{array}{cc} \upsilon_{+}^{\beta }\left[f\;g\right]\left(x\right) & =\upsilon_{+}^{\beta }f\left(x\right)g\left(x\right)+\upsilon_{+}^{\beta }g\left(x\right)f\left(x\right)+{\left[f,g\right]}_{\beta }^{+}\left(x\right),\\ \upsilon_{-}^{\beta }\left[f\;g\right]\left(x\right) & =\upsilon_{-}^{\beta }f\left(x\right)g\left(x\right)+\upsilon_{-}^{\beta }g\left(x\right)f\left(x\right)-{\left[f,g\right]}_{\beta }^{-}\left(x\right), \end{array}$$
- Quotient rule
  $$\begin{array}{cc} \upsilon_{+}^{\beta }\left[f/g\right]\left(x\right) & =\frac{\upsilon_{+}^{\beta }f\left(x\right)g\left(x\right)-\upsilon_{+}^{\beta }g\left(x\right)f\left(x\right)-{\left[f,g\right]}_{\beta }^{+}}{g^{2}\left(x\right)},\\ \upsilon_{-}^{\beta }\left[f/g\right]\left(x\right) & =\frac{\upsilon_{-}^{\beta }f\left(x\right)g\left(x\right)-\upsilon_{-}^{\beta }g\left(x\right)f\left(x\right)+{\left[f,g\right]}_{\beta }^{-}}{g^{2}\left(x\right)}, \end{array}$$

where

$${\left[f,g\right]}_{\beta }^{\pm }\left(x\right):=\lim_{\epsilon \to 0}\upsilon_{\epsilon \pm }^{\gamma }\left[f\right]\left(x\right)\;\upsilon_{\epsilon \pm }^{\beta -\gamma }\left[g\right]\left(x\right),$$

wherever ${\left[f,g\right]}_{\beta }^{\pm }\left(x\right)\neq 0$.

For compositions of functions,

- $f\in H^{\;\beta }$ and $g\in C^{\;1}$
  $$\begin{array}{cc} \upsilon_{+}^{\beta }f\circ g\left(x\right) & =\upsilon_{+}^{\beta }f\left(g\right){\left(g^{\prime }\left(x\right)\right)}^{\beta },\\ \upsilon_{-}^{\beta }f\circ g\left(x\right) & =\upsilon_{-}^{\beta }f\left(g\right){\left(g^{\prime }\left(x\right)\right)}^{\beta }, \end{array}$$
- $f\in C^{\;1}$ and $g\in H^{\;\beta }$
  $$\begin{array}{cc} \upsilon_{+}^{\beta }f\circ g\left(x\right) & =f^{\prime }\left(g\right)\;\upsilon_{+}^{\beta }g\left(x\right),\\ \upsilon_{-}^{\beta }f\circ g\left(x\right) & =f^{\prime }\left(g\right)\;\upsilon_{-}^{\beta }g\left(x\right). \end{array}$$

Basic evaluation formula [51]:

$$\upsilon_{\pm }^{\beta }f\left(x\right)=\frac{1}{\beta }\lim_{\epsilon \to 0}\epsilon^{1-\beta }f^{\prime }\left(x\pm \epsilon \right).$$

Derivative regularization [52]:

Let $f\left(t,w\right)\in C^{2}$ be composition with w(x), a 1/q-differentiable function at *x*, then

(A3) $$\frac{d^{\pm }}{dx}f\left(x,w\right)=\frac{\partial f}{\partial x}+\frac{d^{\pm }}{dx}w\left(x\right)\cdot \frac{\partial f}{\partial w}\pm \frac{1}{q!}{\left[w^{q}\right]}^{\pm }\cdot \frac{\partial^{q}f}{\partial w^{q}},$$

where

$${\left[w^{q}\right]}^{\pm }={\left(\upsilon_{\pm }^{1/q}w\left(x\right)\right)}^{q}$$

is the fractal *q*-adic (co-)variation.

## Appendix B. The Stochastic Variation Problem

The study of stochastic Lagrangian variational principles has been motivated initially by quantum mechanics and optimal control problems. This section gives only a sketch for the treatment of the problem. The reader is directed to [21,30,31] for more details. In the simplest form, this is the minimization of the regularized functional assuming a constant diffusion coefficient *b*

$$S_{\alpha }\left(t_{0},T\right):=\lim_{N\to \infty }E\left(\left.\left(P_{N}\right)\sum_{t=t_{0}}^{t=T}\frac{1}{2}\frac{{\left(\Delta X_{k}\right)}^{2}}{\Delta t_{k}}-\sigma \left(\alpha -\frac{1}{2}\right)b^{2}\right|X_{k}=x\left(\alpha t_{k}+\left(1-\alpha \right)t_{k+1}\right)\right)$$

for the partition $P_{N}$ and $\sigma =\mathrm{sign}\left(\alpha -\frac{1}{2}\right)$.

Thus, suppose that α=1. Then, the increments can be interpreted as Itô integrals so that by the Itô isometry since finite summation and integration commute

$$\begin{array}{c} E\left(\left.\frac{1}{2\Delta t_{k}}{\left(\Delta X_{k}\right)}^{2}-\frac{1}{2}b^{2}\right|X_{k}=x\left(t_{k}\right)\right)=\\ \frac{1}{2\Delta t_{k}}{\left(\int_{t_{k}}^{t_{k+1}}ads\right)}^{2}+\frac{1}{\Delta t_{k}}\left(\int_{t_{k}}^{t_{k+1}}ads\right)E\left(\int_{t_{k}}^{t_{k+1}}bdw\right)+\frac{1}{2\Delta t_{k}}E{\left(\int_{t_{k}}^{t_{k+1}}bdw\right)}^{2}-\frac{1}{2}b^{2}=\\ \frac{1}{2\Delta t_{k}}{\left(\int_{t_{k}}^{t_{k+1}}ads\right)}^{2}+\frac{1}{2\Delta t_{k}}\int_{t_{k}}^{t_{k+1}}b^{2}ds-\frac{1}{2}b^{2}=a\int_{t_{k}}^{t_{k+1}}ads+O\left(\Delta t_{k}\right). \end{array}$$

Therefore, $S_{\alpha }\left(t_{0},T\right)$ is minimal if the drift vanishes on $P_{N}$. Suppose that $X_{t}$ is varied by a small smooth function λϕ(t,x), where the smallness is controlled by λ, then the Itô lemma should be applied so that $E(d\delta X_{t}|F)=0$ on the difference process $\delta X_{t}=\lambda \phi \left(t,x\right)dt+bdW_{t}$. Therefore,

(A4) $$E\left(d\phi |F\right)=\lambda dt\left(\frac{\partial }{\partial t}\phi +\phi \frac{\partial }{\partial x}\phi +\frac{b^{2}}{2}\frac{\partial^{2}}{\partial x^{2}}\phi \right)=0$$

should hold. The same calculation can be performed for α=0 if the Itô integral is replaced by the anticipative Itô integral. In this case, σ=−1 and the integration is reversed

$$\begin{array}{c} E\left(\left.\frac{1}{2\Delta t_{k}}{\left(\Delta X_{k}\right)}^{2}+\frac{1}{2}b^{2}\right|X_{k}=x\left(t_{k+1}\right)\right)=\\ \frac{1}{2\Delta t_{k}}{\left(\int_{t_{k+1}}^{t_{k}}ads\right)}^{2}+\frac{1}{\Delta t_{k}}\left(\int_{t_{k+1}}^{t_{k}}ads\right)E\left(\int_{t_{k+1}}^{t_{k}}bdw\right)+\frac{1}{2\Delta t_{k}}E{\left(\int_{t_{k+1}}^{t_{k}}bdw\right)}^{2}+\frac{1}{2}b^{2}=\\ \frac{1}{2\Delta t_{k}}{\left(\int_{t_{k+1}}^{t_{k}}ads\right)}^{2}+\frac{1}{2\Delta t_{k}}\int_{t_{k+1}}^{t_{k}}b^{2}ds+\frac{1}{2}b^{2}=a\int_{t_{k}}^{t_{k+1}}ads+O\left(\Delta t_{k}\right). \end{array}$$

In this case, the backward Itô formula also applies as

(A5) $$E\left(d\phi |F\right)=\lambda dt\left(\frac{\partial }{\partial t}\phi +\phi \frac{\partial }{\partial x}\phi -\frac{b^{2}}{2}\frac{\partial^{2}}{\partial x^{2}}\phi \right)=0.$$

**Remark** **A1.** The treatment of Pavon [21] uses the symmetrized functional $S=S_{0}+S_{1}$ together with a constraint on anti-symmetrized functional $S_{0}-S_{1}$ in the present notation.

## Appendix C. Matlab Simulation Code

Listing 1: Exact simulation Matlab code.
